# 维奈克拉联合阿扎胞苷治疗不适合标准化疗的新诊断急性髓系白血病疗效分析：单中心数据

**DOI:** 10.3760/cma.j.issn.0253-2727.2022.10.005

**Published:** 2022-10

**Authors:** 立 孙, 少杰 叶, 楠 周, 新智 韩, 佳旭 齐, 小军 刘, 建民 罗, 琳 杨

**Affiliations:** 河北医科大学第二医院血液科，河北省血液病学重点实验室，石家庄 050000 Department of Hematology, the Second Hospital of Hebei Medical University, Key Laboratory of Hematology, Shijiazhuang 050000, China

**Keywords:** 白血病，髓系，急性, 维奈克拉, 抗肿瘤联合化疗方案, Leukemia, myeloid, acute, Venetoclax, Antineoplastic combined chemotherapy protocols

## Abstract

**目的:**

回顾性分析维奈克拉联合阿扎胞苷（venetoclax+ azacitidine，VA）治疗不适合标准化疗的新诊断急性髓系白血病（AML）患者的疗效和安全性。

**方法:**

收集河北医科大学第二医院自2020年5月至2022年3月收治的VA方案治疗的66例不适合标准化疗新诊断AML患者的临床资料，回顾性分析其完全缓解（CR）率、总反应率（ORR）、微小残留病（MRD）阴性率、无事件生存（EFS）率、总生存（OS）率及不良反应发生率。比较不同年龄、ECOG评分、原发与继发、预后分层和分子突变亚组患者的疗效和生存差异。

**结果:**

中位随访4.25（0.9～19.9）个月，中位疗程数2（1～8）个。1个疗程复合CR（cCR）率［CR+血液学未完全恢复的CR（CRi）率］和MRD阴性率分别为78.8％、51.9％，≥2个疗程的cCR率和MRD阴性率分别为81.8％、66.7％。中位EFS和OS时间分别为13.2、15.3个月。亚组分析显示，继发AML患者疗效及生存均差于原发AML患者，发生反弹性血小板增多患者疗效及生存显著优于无反弹性血小板增多患者（*P*值均<0.05）。IDH1/2突变及NPM1突变患者1个疗程CR率均显著高于无相应突变组，存在表观遗传学修饰相关DAT（DNMT3、ASXL1、TET2）突变的患者，≥2个疗程的持续治疗更可能获得最佳疗效。最常见的3～4级不良反应为中性粒细胞减少、血小板减少和贫血。

**结论:**

真实世界不适合标准化疗的新诊断AML患者应用VA方案可较快获得深层次缓解。原发AML、IDH1/2突变、NPM1突变及反弹性血小板增多是治疗获益的有利因素。不良反应可耐受。

## Abstract (en)

**Objective:**

To investigate the effectiveness and safety of the VA regimen, which combines venetoclax with azacitidine in the treatment of patients with newly diagnosed acute myeloid leukemia（AML）who are not suitable candidates for conventional chemotherapy.

**Methods:**

In the Department of Hematology at the Second Hospital of Hebei Medical University, 66 AML patients who received venetoclax and azacitidine treatment from May 2020 to March 2022 were the subject of a retrospective study. The complete remission（CR）rate, cCR rate, ORR rate, MRD negative rate, the incidence of adverse events,1-year EFS, and OS were retrospectively analyzed. Patients subgroups with varying ages, ECOG scores, primary and secondary, risk stratifications, and gene mutation were compared for differences in efficacy and survival.

**Results:**

The median follow-up was 4.25（0.9–19.9）months, and the median number of treatment courses was 2（1–8）cycles. After the first cycle, the cCR rate was 78.8％, and the MRD negative rate was 51.9％. After prolonged treatment, the cCR rate was 81.8％ and MRD negative rate was 66.7％. The median EFS and OS, respectively, were13.2 and 15.3 months. Secondary AML showed inferior efficacy and prognosis. IDH1/2 or NPM1 mutation groups had a significantly higher rate of CR than the control group（*P*<0.05）. The CR rate and MRD negative rate of patients with rebound thrombocytosis were significantly higher than those without rebound thrombocytosis（*P*<0.05）. Those who had epigenetic modification mutations（DNMT3, ASXL1, TET2）were more likely to benefit from ongoing therapy. The most common grade 3 and 4 adverse reactions were neutropenia, thrombocytopenia, and anemia.

**Conclusion:**

In real-world patients with newly diagnosed AML who are not candidates for standard chemotherapy, the VA regimen produces rapid deep remission. Primary AML patients, rebound thrombocytosis, IDH1/2, and NPM1 gene mutations are favorable factors for treatment benefit, and adverse reactions were tolerable.

维奈克拉（venetoclax，VEN）联合去甲基化药物方案已经成为年龄≥75岁或不适合标准化疗的新诊断急性髓系白血病（AML）患者的一线治疗[1]，此方案在中国AML患者中的疗效及安全性尚未得到大规模真实世界研究证实。本研究回顾性分析了66例VEN联合阿扎胞苷（AZA）即VA方案治疗不适合标准化疗的新诊断AML患者的疗效及安全性，以期为临床实践提供指导。

## 病例与方法

1. 病例：收集2020年5月1日到2022年3月1日就诊于河北医科大学第二医院血液内科的66例不适合标准化疗[2]的新诊断AML（非急性早幼粒细胞白血病）患者临床资料，所有患者的AML诊断均符合文献[1]标准，均应用VA方案为主的诱导化疗。

2. 治疗方案: 患者应用VA方案[3]诱导化疗，VEN 100 mg 第1天，200 mg 第2天，400 mg 第3～28天；AZA 75 mg·m^−2^·d^−1^，第1～7天，28 d为1个疗程。诱导化疗第14或第21天行骨髓穿刺评估病情，若患者发生4级中性粒细胞减少或4级血小板减少，存在严重感染或出血风险，在骨髓原始细胞≤5％情况下停用VEN。停用VEN第14天检测中性粒细胞绝对计数（ANC）仍<1.5×10^9^/L，或PLT仍<100×10^9^/L（血象恢复延迟）的患者，等待ANC≥0.5×10^9^/L，且PLT≥50×10^9^/L时再开始下一疗程VA方案治疗，在下一疗程VA方案中VEN治疗天数较前减少7 d[4]；若VEN治疗天数减少至7 d，患者仍存在血象恢复延迟，不再缩短VEN治疗天数，将VA方案中AZA治疗天数由7 d缩短为5 d（75 mg·m^−2^·d^−1^）；若VA方案调整为VEN 400 mg 第1～7天联合AZA 75 mg·m^−2^·d^−1^ 第1～5天后，患者仍存在血象恢复延迟，调整为AZA单药（75 mg·m^−2^·d^−1^ 第1～5天）方案与VEN（400 mg 第1～7天）联合AZA（75 mg·m^−2^·d^−1^ 第1～5天）方案交替应用。在每次化疗前评估患者综合情况，若患者后期动态评估显示适合标准化疗，可调整为标准化疗方案进行巩固治疗。

3. 疗效及不良反应判定：依据2017年欧洲白血病工作组指南[5]进行疗效评价，包括完全缓解（CR）、CR伴血液学不完全恢复（CRi）、形态学无白血病状态（MLFS）、部分缓解（PR）、复合完全缓解（cCR）、总反应率（ORR），其中cCR为CR+CRi，ORR为CR率+CRi率+MLFS率+PR率。反弹性血小板增多定义为在化疗后出现血小板恢复正常后反弹升高至>400×10^9^/L。依据NCI CTCAE 5.0[6]进行不良反应评价。肿瘤溶解综合征（TLS）依据Cairo-Bishop标准[7]定义。合并症评价采用年龄校正的Charlson合并症评分[8]。

4. 微小残留病（MRD）的检测方法：髓溶红细胞法制备骨髓液标本，在2个流式管中以九色法进行标记（第1管：CD38/CD117/CD123/CD34/CD33/CD13/ HLA-DR/CD11b/CD45，第2管：CD15/CD34/CD56/CD33/CD7/CD14/CD19/CD45），应用NAVIOS流式细胞仪（美国贝克曼库尔特公司产品）检测，至少检测500 000个有核细胞。参考2018 欧洲白血病网（ELN）发布的MRD监测指南[9]，MRD>0.1％（占WBC）定义为阳性。

5. 随访：通过查阅电子病历、门诊随诊、电话随访等方式对患者的生存和复发状态进行随访。末次随访时间为2022年3月17日。总生存（OS）率及无事件生存（EFS）率根据2017年欧洲白血病工作组指南[5]定义。

6. 统计学处理：依据SPSS26.0软件进行统计学分析，计数资料以百分比（％）表示，应用卡方检验及Fisher确切概率法进行差异性检验；生存分析应用Kaplan-Meier法绘制生存曲线，应用Log-rank法进行单因素检验，检验水准*α*＝0.05，*P*<0.05为差异有统计学意义。

## 结果

1. 患者的基线特征：66例AML患者中位发病年龄65（32～82）岁，男35例，女31例，患者治疗前临床特征见表1。

**表1:** 66例新诊断急性髓系白血病患者的基线特征

特征	数值
年龄［岁，*M*（范围）］	65（32～82）
诊断时骨髓原始细胞［％，*M*（范围）］	56.0（20.0～96.5）
疾病类型［例（％）］	
原发性	50（75.8）
继发性	16（24.2）
性别［例（％）］	
男	35（53.0）
女	31（47.0）
ECOG评分［例（％）］	
0～1	33（50.0）
2～4	33（50.0）
ELN危险度分层［例（％）］	
低危	20（30.3）
中危	22（33.3）
高危	24（36.4）
基因突变［例（％）］	
IDH1/2	23（34.8）
FLT3-ITD/TKD	21（31.8）
DNMT3A	21（31.8）
K/N-RAS	16（24.2）
TET2	14（21.2）
NPM1	13（19.7）
ASXL1	12（18.2）
CEBPA	9（13.6）
RUNX1	7（10.6）
TP53	4（6.1）
JAK2	2（3.0）

注：ECOG：美国东部肿瘤协作组；ELN：欧洲白血病网

在VEN治疗天数调整方面，第1疗程化疗中，因出现4级中性粒细胞减少和（或）4级血小板减少同时合并严重感染和（或）出血风险，13例（19.7％）VEN疗程减为21 d，8例（12.1％）VEN减为14 d，仅45例（68.2％）VEN用足28 d；第2疗程及其后的化疗阶段，截至末次随访，有35例（53.0％）患者因血象恢复延迟经历减低剂量的调整，其中7例（20.0％）VEN减为21 d，18例（51.4％）VEN减为14 d，3例（8.6％）VEN减为7 d，7例（20.0％）调整为VA方案（VEN 400 mg第1～7天联合AZA 75 mg·m^−2^·d^−1^第1～5天）和AZA（75 mg·m^−2^·d^−1^，第1～5天）交替应用。

2. 疗效评价：1个疗程VA方案治疗的ORR、cCR率、CR率和MRD阴性率分别为87.9％、78.8％、56.1％和51.9％。≥2个疗程的ORR、cCR、CR率和MRD阴性率分别为90.9％、81.8％、65.2％和66.7％。最佳反应达到CR的43例患者中，37例（86％）为1个疗程治疗后获得CR；4例（9.4％）为第1疗程首先获得CRi，2个疗程治疗后达CR；1例（2.3％）第1疗程未缓解，2个疗程治疗后CR；1例（2.3％）第1疗程获得MLFS，3个疗程治疗后获得最佳反应CR。中位应答时间为1（0.6～3.1）个月，中位反应持续时间为12.2（9.2～15.2）个月。

我们进一步分析了不同亚组患者的疗效，结果显示原发AML患者1个疗程的cCR率和CR率以及≥2个疗程获得的CR率显著高于继发性AML患者（*P*值均<0.05）。不同ELN预后分层、年龄（≥75岁和<75岁）、ECOG评分患者的1个疗程及≥2个疗程CR率、cCR率、ORR、MRD阴性率差异均无统计学意义（*P*值均>0.05）（表2）。诱导化疗VEN应用天数（14 d、21 d和28 d）对1个疗程CR率、cCR率、ORR和MRD阴性率无显著影响（62.5％对46.2％对57.8％，100.0％对69.2％对77.8％，100.0％对76.9％对 88.9％，50.0％对77.8％ 对45.7％，*P*值均>0.05）。对于≥2个疗程治疗后因血象恢复延迟缩短VEN疗程的患者，目前完成疗程数较少，暂不能比较缩短VEN应用时间对疗效、生存及复发的影响。

我们对突变例数大于10例的亚组的疗效进行统计分析，IDH1/2突变组和NPM1突变组1个疗程CR率均较无相应突变组显著升高（*P*＝0.033，*P*＝0.003）。有无FLT3突变、K/N-RAS突变患者在1个疗程及≥2个疗程CR率、cCR率、ORR和MRD阴性率方面差异均无统计学意义（*P*值均>0.05）（表2）。

**表2:** 66例急性髓系白血病（AML）不同亚组间疗效的比较［例（％）］

组别	例数	1个疗程疗效				最佳疗效			
		CR	cCR	ORR	cCR患者中MRD阴性	CR	cCR	ORR	cCR患者中MRD阴性
AML类型									
原发	50	32(64.0)	43(86.0)	46(92.0)	24(55.8)	36(72.0)	44(88.0)	47(94.0)	29(65.9)
继发	16	5(31.3)^a^	9(56.3)^a^	12(75.0)	3(33.3)	7(43.8)^a^	10(62.5)	13(81.3)	7(70.0)
年龄									
≥75岁	9	5(55.6)	8(88.9)	9(100.0)	3(37.5)	7(77.8)	8(88.9)	9(100.0)	5(62.5)
<75岁	57	32(56.1)	44(77.2)	49(86.0)	24(54.5)	36(63.2)	46(80.7)	51(89.5)	31(67.4)
ECOG评分									
0~1	33	19(57.6)	23(69.7)	27(81.8)	14(60.9)	23(69.7)	25(75.8)	29(87.9)	20(80.0)
2~4	33	18(54.5)	29(87.9)	31(93.9)	13(44.8)	20(60.6)	29(87.9)	31(93.9)	16(55.2)
ELN分层									
低危	20	13(65.0)	17(85.0)	18(90.0)	11(64.7)	16(80.0)	18(90.0)	18(90.0)	12(66.7)
中危	22	15(68.2)	19(86.4)	21(95.5)	9(47.4)	15(68.2)	19(86.4)	21(95.5)	13(68.4)
高危	24	9(37.5)	16(66.7)	19(79.2)	7(43.8)	12(50.0)	17(70.8)	21(87.5)	11(64.7)
反弹性血小板增多									
是	15	13(86.7)	15(100.0)	15(100.0)	12(80.0)	15(100.0)	15(100.0)	15(100.0)	15(100.0)
否	51	23(45.1)^b^	37(72.5)^a^	43(84.3)	15(40.5)^a^	27(52.9)^b^	39(76.5)	45(88.2)	20(51.3)^b^
分子遗传学特征									
IDH1/2突变									
是	23	17(73.9)	21(91.3)	22(95.7)	13(61.9)	18(78.3)	21(91.3)	22(95.7)	16(76.2)
否	43	17(39.5)^a^	29(67.4)	34(79.1)	14(48.3)	22(51.1)	31(72.1)	36(83.7)	19(61.3)
FLT3突变									
是	21	14(66.7)	17(81.8)	19(90.5)	8(47.1)	16(76.2)	18(85.7)	20(95.2)	14(77.8)
否	45	21(46.7)	33(73.3)	38(84.4)	19(57.5)	25(75.6)	34(75.6)	39(86.7)	22(64.7)
DNMT3A突变									
是	21	15(71.4)	19(90.6)	20(95.2)	12(63.2)	17(81.0)	19(90.5)	20(95.2)	17(89.5)
否	45	19(42.2)	31(68.9)	37(82.2)	15(48.4)	23(51.1)	33(73.3)	39(86.7)	20(60.6)^a^
K/N-RAS突变									
是	16	8(50.0)	12(75.0)	14(87.5)	8(66.7)	9(56.3)	12(75.0)	14(87.5)	9(75.0)
否	50	25(50.0)	37(74.0)	41(82.0)	19(51.4)	30(60.0)	39(78.0)	43(86.0)	26(66.7)
TET2突变									
是	14	6(42.9)	9(64.3)	10(71.4)	5(55.6)	7(50.0)	9(64.3)	12(85.7)	7(77.8)
否	52	28(53.8)	31(59.6)	36(69.2)	17(54.8)	33(63.5)	33(63.5)	36(69.2)^b^	21(63.6)
NPM1突变									
是	13	12(92.3)	13(100.0)	13(100.0)	8(61.5)	13(100.0)	13(100.0)	13(100.0)	10(76.9)
否	53	22(41.5)^b^	37(69.8)	43(81.1)	19(51.4)	27(50.9)^b^	39(73.6)	45(84.9)	26(66.7)
ASXL1突变									
是	12	4(33.3)	8(66.7)	8(66.7)	5(62.5)	5(41.7)	8(66.7)	9(75.0)	6(75.0)
否	54	29(53.7)	41(75.9)	47(87.0)^a^	22(53.7)	34(63.0)	43(79.6)	48(88.9)	29(67.4)

注：CR：完全缓解；cCR：复合完全缓解（CR+CR伴血液学不完全恢复）；ORR：总体反应率；MRD：微小残留病；ECOG：美国东部肿瘤协作组；ELN：欧洲白血病工作组。两组比较，^a^ *P*<0.05，^b^*P*<0.01

DNMT3A突变组≥2个疗程MRD阴性率较无DNMT3突变组更高（*P*＝0.009）。ASXL1突变组的1个疗程ORR显著低于无ASXL1突变组（*P*<0.05），但随着疗程数增加，两组间差异消失。TET2突变组的≥2个疗程ORR显著高于无TET2突变患者（*P*<0.05）。提示我们对于存在表观遗传学修饰相关的DAT（DNMT3A、ASXL1、TET2）突变的患者，≥2个疗程的持续治疗更可能获得最佳疗效。

有22.7％（15/66）的患者首个疗程治疗中出现反弹性血小板增多，首个疗程均获得CR/CRi的疗效，疗程数增加后15例患者全部获得CR。出现反弹性血小板增多的患者CR率和MRD阴性率均明显高于无反弹性血小板增多患者（*P*<0.05），截至随访截止日期，该亚组所有患者均处于持续缓解状态。

3. 生存分析：中位随访时间为4.25（0.9～19.9）个月，总体的中位EFS时间为13.2（3.7～22.7）个月，中位OS时间为15.3（0.9～19.9）个月（图1）。共死亡9例，其中30 d内死亡1例，系合并重症胰腺炎的42岁男性，死于消化道出血所致失血性休克，30 d死亡率1.5％。60 d内死亡2例，另1例为72岁男性患者，诊断时存在肺间质纤维化，死于重症肺炎呼吸衰竭，60 d死亡率3.0％。14例患者获得CR后评估综合情况适合标准化疗，调整为中大剂量阿糖胞苷方案巩固治疗，2例患者桥接异基因造血干细胞移植，截至随访截止，除1例患者死亡外，其余患者均处于无复发生存中。CR患者的中位EFS及OS时间明显长于未达CR患者（*P*均<0.001）（图2）。cCR患者及MRD阴性患者的中位EFS时间显著长于未达cCR患者（*P*<0.001，*P*＝0.039），出现反弹性血小板增多患者的中位EFS时间较无反弹性血小板增多患者显著延长（*P*＝0.013），原发AML较继发AML患者的中位EFS时间更长（*P*＝0.011）（图3）。NPM1突变亚组、DNMT3A突变亚组中位EFS时间较无相应突变亚组延长：有无NPM1突变两组中位EFS 时间分别为未达到和 12.1个月（*P*＝0.024），有无DNMT3A突变两组中位EFS时间分别为未达到和 12.1个月（*P*＝0.039）。TET2突变亚组相比无相应突变亚组的中位EFS时间明显缩短：有无TET2突变两组中位EFS 时间分别为 3.4个月和 13.2个月（*P*＝0.025）。

**图1:**
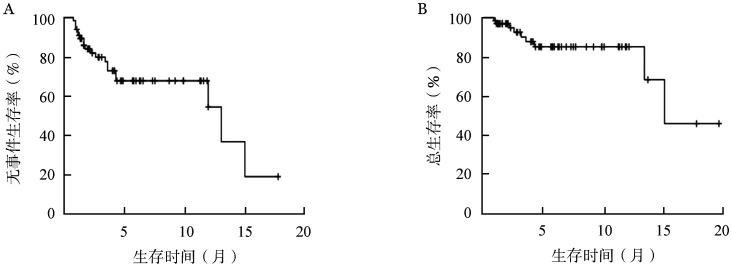
66例接受维奈克拉联合阿扎胞苷方案治疗的新诊断不适合标准化疗急性髓系白血病患者的无事件生存（A）及总生存曲线（B）

**图2:**
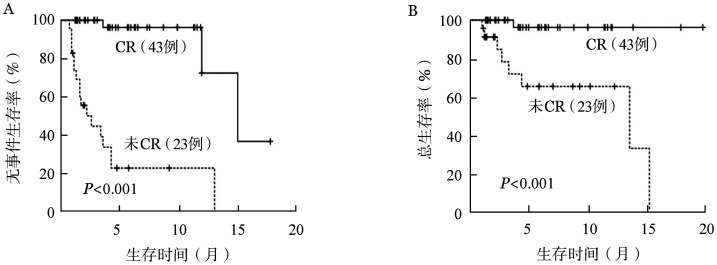
获得完全缓解（CR）与未获得CR组的无事件生存（A）和总生存（B）比较

**图3:**
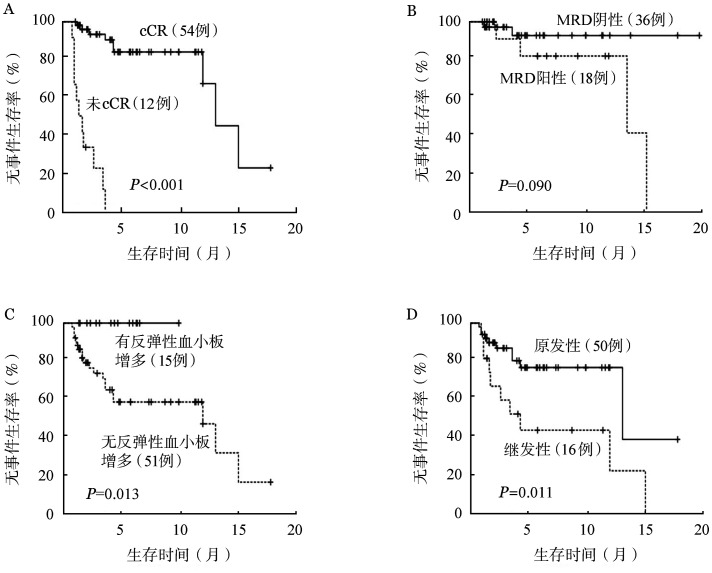
维奈克拉联合阿扎胞苷方案治疗新诊断不适合标准化疗急性髓系白血病患者不同亚组的无事件生存比较 A：复合完全缓解（cCR）状态；B：微小残留病（MRD）状态；C：反弹性血小板增多；D：疾病类型

4. 不良反应：最常见的3～4级不良反应为血液系统的中性粒细胞减少、血小板减少和贫血。65例患者存在3～4级粒细胞减少，其中46例（70.8％）在治疗前即存在，19例（29.2％）在治疗期间出现，中位中性粒细胞恢复时间为31（0～94）d。61例患者存在3～4级血小板减少，其中41例（67.2％）在治疗前即存在，20例（32.8％）在治疗期间出现，中位血小板恢复时间为27（2～94）d。60例患者存在3～4级贫血，其中45例在治疗前即存在，15例（25.0％）在治疗期间出现。62.1％（41/66）的患者发生粒细胞减少性发热。

非血液学不良反应中最常见的为感染，59.1％（39例）的患者出现感染，其中肺部感染35例（53.0％），血流感染6例，泌尿系感染2例，肛周感染3例，肠道感染1例，腹腔感染1例；其次为胃肠道不良反应，30例患者出现恶心，25例出现呕吐，10例出现腹泻，15例出现便秘；13例患者出现肝功能损伤，大部分为轻度，仅1例患者发生重度肝损伤。监测到22.7％（15例）的患者出现实验室TLS，未出现临床TLS。8例患者发生出血性事件，包括1例消化道出血，1例脑出血，1例肺出血，3例泌尿系统出血，2例鼻出血。3例发生血栓性事件，均为轻度。

## 讨论

与VIALE-A临床研究相比，本研究66例患者最佳疗效的cCR率达到81.8％，MRD阴性率达66.7％，均优于VIALE-A研究[3]中66.4％的cCR率及23.4％的MRD阴性率，VIALE-A研究[3]仅入组了ECOG评分0～1分患者，然而在真实世界中，存在着大量因各种合并症导致体能状况差、ECOG评分≥2分的患者群体，本队列有50％的患者ECOG评分2～4分，该组的cCR率为87.9％，MRD阴性率为55.2％，与ECOG评分0～1分组在疗效和生存方面差异均无统计学意义。获得MRD阴性的AML患者较MRD阳性患者有更长的EFS及OS时间[10]。VA方案在本中心的临床实践中使患者较快速获得深度缓解，在体能状况差、ECOG评分2～4分的AML患者中仍能取得较好疗效，可能转化为更好的生存获益。

本研究中反弹性血小板增多患者的CR率、MRD阴性率及EFS均明显优于无反弹性血小板增多患者，OS亦有延长趋势，在反弹性血小板增多组中存在IDH1/2突变的患者较无反弹性血小板增多组更多（53.3％对29.4％），这一结果与文献报道一致：Schnell等 [11]分析了291例标准化疗的AML患者，41.2％出现了反弹性血小板增多（>500×10^9^/L）；多因素分析示反弹性血小板增多是长期生存的独立影响因素，与诊断时低血小板水平、高比例骨髓浸润、NPM1突变、ELN低危相关。Othman 等[12]分析了123例接受VA方案的初治AML患者，22.7％出现了反弹性血小板增多（>400×10^9^/L），发生反弹性血小板增多的患者CR/CRi率高，有更长的OS时间趋势，伴有IDH1/2突变比例高。

FLT3突变AML患者预后差，复发率高，生存期短[13]。Maiti等[10]报道VA方案在FLT3突变患者中取得72％的cCR率，与无FLT3突变亚组比较，差异无统计学意义。本研究中合并FLT3突变亚组的cCR率为85.7％，与无FLT3突变组的CR率、MRD阴性率、EFS、OS差异均无统计学意义，提示与标准化疗方案比较，VA方案有可能克服FLT3突变相关的不良预后影响。Maiti等[14]探索了FLT3i-DEC10-VEN三药联合方案在FLT3突变的初治AML患者中的疗效和安全性，结果显示PCR/二代测序检测的MRD阴性率为91％。本研究1例患者在诱导阶段联合索拉非尼，1例巩固阶段单用索拉非尼，均获得了缓解，且未增加新的不良反应。Kim等[15]报道DEC10-VEN方案在TP53突变患者中的ORR、cCR率、中位OS和RFS均明显劣于无TP53突变亚组。本研究中4例TP53突变患者有3例获得cCR，与无TP53突变组在cCR率、EFS和OS方面差异无统计学意义，但该3例获得缓解患者中2例同时存在IDH1/2突变，可能是其疗效优于文献报道的原因之一。本研究中TP53突变亚组分析中的患者数较少，结论尚需更多研究证实。

安全性方面，VIALE-A研究[3]试验组中45％的患者出现了≥3级的血小板减少，42％的患者出现了≥3级的中性粒细胞减少，84％的患者出现了不同程度的感染。本队列中血液学不良反应的发生率更高，与真实临床实践中患者的ECOG评分更高、有更高比例伴有合并症相关。22.7％的患者出现了实验室TLS，未出现临床TLS，低于文献报道[16]。30 d和60 d内死亡率分别为1.5％和3.0％，远低于文献报道的标准化疗治疗老年AML的早期死亡率[17]。此外本中心数据显示第1疗程化疗中，21例（31.8％）患者VEN应用天数减为21 d或14 d，仅45例（68.2％）患者VEN应用28 d；在第2疗程及其后的化疗阶段，有35例（53％）患者因血象恢复延迟减少VEN应用天数，考虑与老年AML患者正常造血功能储备较差相关。初步分析显示诱导化疗期间VEN应用天数对首疗程疗效无显著影响，但后续VEN应用天数减少对维持缓解、复发及生存的影响仍需要扩大样本数量及更长期的随访观察来判定。

总之，本研究结果显示VA方案一线应用治疗年龄≥75岁或不适合标准化疗的新诊断AML患者可取得较快的深层次缓解，表现出良好安全性。亚组分析中，IDH1/2突变、NPM1突变及出现反弹血小板增多是VA方案治疗获益的积极影响因素，我们将进一步扩大样本量研究不同的分子遗传学特征在VA方案治疗选择中的意义，以及维持治疗期间VEN治疗天数对患者疗效和长期生存的影响。
